# Automated processing of label-free Raman microscope images of macrophage cells with standardized regression for high-throughput analysis

**DOI:** 10.1186/1745-7580-6-11

**Published:** 2010-11-19

**Authors:** Robert J Milewski, Yutaro Kumagai, Katsumasa Fujita, Daron M Standley, Nicholas I Smith

**Affiliations:** 1 Laboratory of Systems Immunology, WPI Immunology Frontier Research Center (IFReC), Osaka University, 3-1 Yamadaoka, Suita, Osaka 565-0871, Japan; 2 Laboratory of Host Defense, WPI Immunology Frontier Research Center (IFReC), Osaka University, 3-1 Yamadaoka, Suita, Osaka 565-0871, Japan; 3 Dept Applied Physics, Osaka University, 2-1 Yamadaoka, Suita, Osaka 565-0871, Japan; 4 Precursory Research for Embryonic Science and Technology (PRESTO), Japan Science and Technology Agency (JST), Sanbancho 3-5, Chiyodaku, Tokyo, 102-0075; 5 Biophotonics Laboratory, WPI Immunology Frontier Research Center (IFReC), Osaka University, 3-1 Yamadaoka, Suita, Osaka 565-0871, Japan

## Abstract

**Background:**

Macrophages represent the front lines of our immune system; they recognize and engulf pathogens or foreign particles thus initiating the immune response. Imaging macrophages presents unique challenges, as most optical techniques require labeling or staining of the cellular compartments in order to resolve organelles, and such stains or labels have the potential to perturb the cell, particularly in cases where incomplete information exists regarding the precise cellular reaction under observation. Label-free imaging techniques such as Raman microscopy are thus valuable tools for studying the transformations that occur in immune cells upon activation, both on the molecular and organelle levels. Due to extremely low signal levels, however, Raman microscopy requires sophisticated image processing techniques for noise reduction and signal extraction. To date, efficient, automated algorithms for resolving sub-cellular features in noisy, multi-dimensional image sets have not been explored extensively.

**Results:**

We show that hybrid z-score normalization and standard regression (Z-LSR) can highlight the spectral differences within the cell and provide image contrast dependent on spectral content. In contrast to typical Raman imaging processing methods using multivariate analysis, such as single value decomposition (SVD), our implementation of the Z-LSR method can operate nearly in real-time. In spite of its computational simplicity, Z-LSR can automatically remove background and bias in the signal, improve the resolution of spatially distributed spectral differences and enable sub-cellular features to be resolved in Raman microscopy images of mouse macrophage cells. Significantly, the Z-LSR processed images automatically exhibited subcellular architectures whereas SVD, in general, requires human assistance in selecting the components of interest.

**Conclusions:**

The computational efficiency of Z-LSR enables automated resolution of sub-cellular features in large Raman microscopy data sets without compromise in image quality or information loss in associated spectra. These results motivate further use of label free microscopy techniques in real-time imaging of live immune cells.

## Background

Raman scattering (additional file 1) is a well-known process that has been studied for decades. The Raman effect has a wide range of potential applications due to its sensitivity to the chemical composition of diverse samples. This sensitivity is now being applied to cellular imaging, although the potential applications of Raman imaging to immunology remain largely unexplored. Recent papers (for example, [1-4]) have shown that diagnosis of cell structure and or cell type is feasible with modern Raman spectroscopic techniques, in a completely label-free and physiologically normal cell environment. However, while the feasibility has been shown, such techniques are not yet widely applied in the immunology field. The reason for this is primarily due to the inherently low signals acquired in Raman imaging. Raman microscopy can be used in combination with metallic probes or tuned to resonant frequencies in the cell [5] to improve signal levels. However, for overall observation of cellular reactions involving potentially unknown molecules and signaling mechanisms, "spontaneous" or label-free Raman microscopy is the least invasive method for acquiring data on immune cell components and dynamics or reactions accompanying the immune response. Using only light scattering as the contrast mechanism, Raman spectroscopy can capture the chemical signature and distributions of molecules characteristic of activation processes in host immune cells, albeit subject to significant restrictions due to signal to noise levels. Label-free Raman microscopy then requires sophisticated image processing techniques for noise reduction and signal extraction [6,7]. Efficient, automated algorithms for resolving sub-cellular features in noisy, multi-dimensional image sets have not been explored extensively in the context of specific immune cell types such as macrophages. Furthermore, in order to become a useful technique in immunology, the image processing techniques must be applicable to automated processing of large data sets.

**Figure 1:**
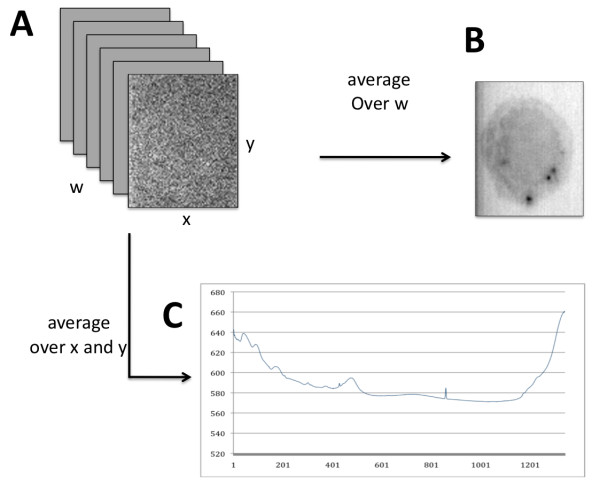
**Raman microscopy data**. The stack of x-y planes (A) can be averaged (over w) to produce a spectrally averaged image of a macrophage cell (B). Conversely, a range of Δx-Δy values can be averaged to produce an overall Raman spectral signal for all locations in the image (C).

As illustrated in figure 1, confocal Raman Microscopy imaging produces a stack (typically thousands) of data planes. Each x-y plane is a spatial map of the intensity at a given spectral position, or wave number, usually measured in inverse centimeters (cm-1) (hereafter referred to as the *w *dimension). Averaging over w-planes reduces noise (figure 1B), but results in a loss of spectral information associated with each x, y point. A trace through all planes of a particular x, y position (or an average over a Δx-Δy area) corresponds to the Raman signal for this region of the specimen (figure 1C).

As mentioned above, individual data planes (corresponding to particular wavenumbers of molecular vibrational frequencies) are dominated by background noise and the cell of interest is often not visible. Simple averaging over the planes produces a much clearer image at the cost of losing spectral information. This motivates further investigation into noise reduction techniques that retain the chemical information inherent in the Raman signals. Complementary to methods for noise reduction are methods for feature extraction based on differences in spectral content within the x-y plane. These differences arise from differences in the abundance of bio-molecules with characteristic vibrational frequencies (i.e., lipids vs. proteins). The ultimate goal in the context of immune cell imaging is to exploit such differences in order to visualize sub-cellular features (i.e., phagosome, mitochondria) or processes (phagocytosis, apoptosis) in live cells without the use of labeling agents that might interfere with such processes.

Because of the high dimensionality of the hyperspectral Raman microscope images, multivariate analysis techniques such as SVD can be employed to reduce the dimensionality to a few key components. The drawback of such approaches is that they are generally computationally expensive, and the computational cost is very sensitive the size of the data set. A less complex way to identify spectral features of interest is to express the raw signal in terms of a z-score. A z-score is a routinely-used dimensionless quantity that expresses raw data in terms standard deviations from the mean. In bio-statistics, for example z-score analysis is often employed in the identification of aberrant tissue samples [8], whereas in bioinformatics, target functions using a particular a scale or units, are often first standardized in terms of z-scores before comparison [9]. In the present study we utilized linear regression of the z-score standardized spectra to weight x, y values of interest (i.e., that deviate significantly from the background). This approach is straightforward, computationally simple, and as we show, effective in pre-processing raw Raman microscopy image sets. To our knowledge, the Z-LSR image processing method proposed in this study has not been used previously for analysis of Raman microscopic images.

## Results and Discussion

### Z-LSR effective for noise reduction and subcellular feature extraction

**Figure 2:**
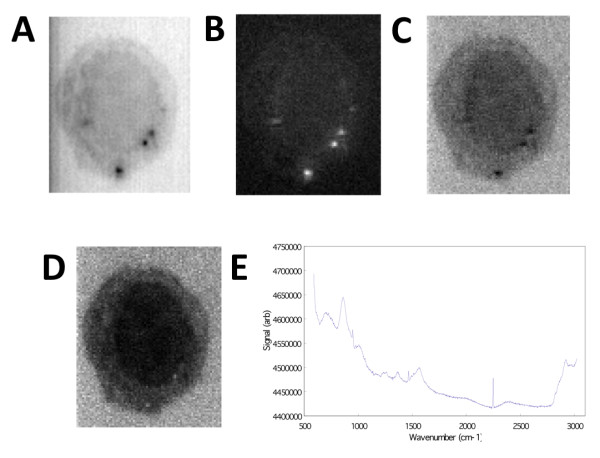
**Generating image contrast by spectral information using Z-LSR**. A) Raw image averaged over the w-axis. B) Regression slopes plotted on the x-y plane. C) The average over all planes of the z-score standardized spectra. D) The re-weighting of the standardized spectra by the regression slopes, showing wavenumber region 2842.6 cm^-1 ^to 3024.5 cm^-1^. E) The normalized, weighted spectrum (averaged over the entire x-y plane) is shown.

We first implemented an automated cosmic ray removal processing step (see Methods section for details) and processed the data to remove such artifacts. The Z-LSR method followed, and was performed in two steps. First, the signals were z-score standardized (equation 1). Second, each x, y coordinate was weighted by the slope of a line fitted to the corresponding standardized (w dimension) Raman signal (equation 2). The final image was constructed by weighting each standardized Raman signal by the corresponding x-y weight. As illustrated in figure 2, the resulting image revealed a distinct cell boundary and some sub-cellular features, such as the nucleus. The method also removed background bias and non-flat field illumination in the image, which can occur when the data of interest is towards the edge of the microscope field of view.

### SVD can determine constituent spectra, extract subcellular features and reduce noise

**Figure 3:**
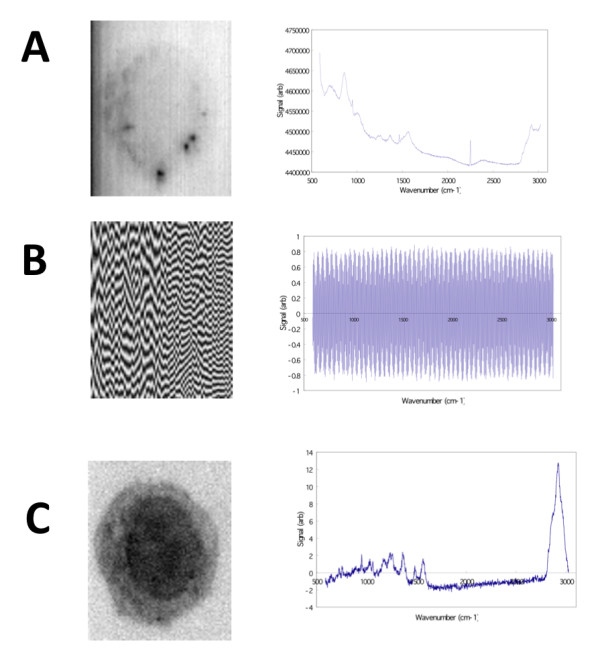
**Image reconstruction (left) for specific SVD eigenvectors (right)**. A) Image reconstructed from the 1^st ^eigenvector reveals a faint outline of the macrophage cell boundary and a non-flat field illumination in the microscope, as well as several dark spots within the cell. B) The 2^nd ^eigenvector corresponds to one component of background noise. C) The reconstructed image using the 3^rd ^eigenvector reveals a distinct macrophage cell boundary and nucleus (showing wavenumber region 2842.6 cm^-1 ^to 3024.5 cm^-1^).

When spectral analysis is required, SVD and reconstruction of the data set by eigenspectra of interest is a computationally costly but powerful method to reduce noise and analyze components in the hyperspectral data. Although used in many application areas, such as computer vision, it has the following drawbacks: (i) both time and space computational complexity is high, resulting in severe scalability issues, (ii) high dimensional computations are difficult for humans to visualize, (iii) in some experiments, there is no eigenvector solution, and (iv) it is possible that SVD produces no clear cellular image even when there is a solution. Computational complexity is costly due mainly to matrix multiplications of order Ο(*N*^2^*M*^2^+*NM*^2^+*M*^3^) such that *N *is the number of matrix rows and *M *is the number of matrix columns. The underlying hypothesis in SVD is that the signal of interest is linearly orthogonal to the noise, thereby allowing the separation of cell structure from noise using the eigenvector(s) of interest. A human can then select the most revealing image using both the spectra and eigenvector-reconstructed image. It is generally expected that the eigenvectors of interest will be those with the highest, or rather, most dimensionally discriminating eigenvalues; however, we typically found that spectral components of interest could be extracted as low as the 20th eigenvalue. An example of 3 eigenvectors is shown in figure 3. Of particular interest is the 3^rd ^eigenvector which shows a clear plasma membrane boundary and nucleus after eigen-reconstruction. The corresponding spectra shows a strong peak in the spectral region 2770-3024 cm^-1^. These eigenspectra show the difficulty of automatic determination of optimal combinations of eigenspectra. Manual inspection is a robust but time-consuming method for assigning signal or noise qualifiers to eigenspectra. We therefore implemented the use of a simple RMSD metric (see Methods section for details) to separate the spectra of interest that can then be used in reconstructing the optimal image.

### Z-LSR is an efficient method for inspection of hyperspectral data

Z-LSR can be used to highlight spectral differences in Raman data by assigning contrast to spatial distributions of spectra. This is often the first step in analysis of acquired data, in order to determine whether the data is of interest. It should be noted that the Z-LSR process is not a competing algorithm with the SVD or multivariate approach. Rather, the Z-LSR is designed for rapid evaluation of large amounts of Raman data. Due to the complexities involved in projecting the hyperspectral data stack into a 2D image, it is not simply a matter of boosting the contrast in the 2D image (which is trivial to automate), but rather finding an automated way to efficiently map the spectral data stack to a grey-scale or false-colored 2D image. Although the computational details are quite different, figures 2 and 3 show that both Z-LSR and SVD methods can produce an image that highlights spectral and spatial distributions.

In order to assess the computational requirements of each method and scalability across diverse data sets, we carried out a series of experiments using 3 different Raman images of macrophage cells that varied in size. Since the cells were living, the data analysis had to be done faster than the image acquisition time, or off-line, after the experiments had been carried out. Table 1 summarizes the results of the 3 selected experimental datasets. The best two eigenvectors for each experiment were selected for comparison using the RMSD method, as described in Methods.

**Table 1:** Showing Raman dataset dimensions, acquisition times, and processing times.

					Z-LSR	SVD
Dataset	Dimensions	Acq. Time	Z-Score	Regr.	Total	
SET1	120 × 400 × 1340	600 s	10 s	10 s	20 s	3.0 h
SET2	81 × 400 × 1340	405 s	5 s	7 s	12 s	1.5 h
SET3	206 × 400 × 1340	1030 s	18 s	15 s	33 s	6.5 h

Processing times include automated cosmic ray removal (see methods sections) for all data sets. Set 1 can be projected to a 120 × 400 image of 2 macrophage cells. Set 2 corresponds to an 81 × 400 pixel image of 2 macrophage cells. Set 3 is a 206 × 400 pixel image of 8 macrophage cells. Times are actual running times on a 2.26 GHZ Quad-Core Intel Xeon 32GB DDR3 Mac Pro

From table 1 it can be readily seen that the Z-LSR method scales linearly with image stack size, whereas SVD and other multivariate methods using matrix multiplications [10] scale nonlinearly with the size of the data. For a single image, such differences might not be a determining factor in selecting the best image processing technique. However, for large-scale automated processing of many image sets, efficiency effectively rules out using such processing. These results suggest that Z-LSR is suitable for automation of large-scale processing Raman microscopic images.

### Z-LSR reveals sub-cellular architecture

**Figure 4:**
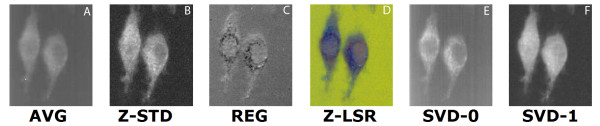
**comparison of set 1**: (a) Average of spectra between 2588 cm^-1^-2861 cm^-1^, (b) Z-score standardization 2915 cm^-1^-3024 cm^-1^, (c) Linear regression, (d) Z-LSR (e) SVD eigenvector 0, (f) SVD eigenvector 1.

**Figure 5:**
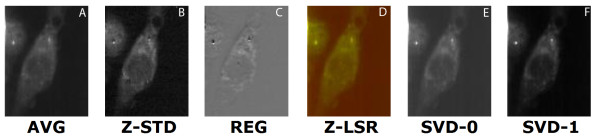
**comparison of set 2**: (a) Average of spectra between 2588 cm^-1^-2861 cm^-1^, (b) Z-score standardization 2915 cm^-1^-3024 cm^-1^, (c) Linear regression, (d) Z-LSR (e) SVD eigenvector 0, (f) SVD eigenvector 1.

**Figure 6:**
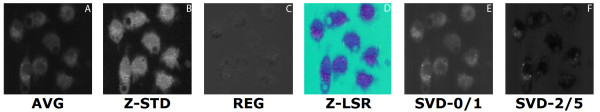
**comparison of set 3**: (a) Average of spectra between 2588 cm^-1^-2861 cm^-1^, (b) Z-score standardization 2915 cm^-1^-3024 cm^-1^, (c) Linear Regression, (d) Z-LSR (e) SVD eigenvectors 0 and 1 are nearly identical (f) SVD eigenvectors 2 and 5 are nearly identical.

Images from the raw data, and Z-LSR reconstructions are shown in figure 4, figure 5, and figure 6. For data sets which show some promising spectra, SVD extracted eigenvectors are also worth considering and are shown for comparison. The Z-LSR automatically produces two intermediate images with high contrast. The Z-score standardization images (following automated cosmic ray noise removal) returned an image which is similar in nature to the raw data but with highlighted contrast in Raman signal from the components inside the cell (see for example, figure 4). The regression image strongly highlights the differential distribution of intracellular components in the cell. The combination of these images can automatically generate strong contrast with no intervention on the part of the user, and is highly suitable for automation and automated comparison of different datasets. The contrast in the final image is not merely a contrast-boosted version of the original data. Rather, the actual contrast mechanism is inherently generated from the differences in spectra within specific regions of the cell. The Z-LSR image additionally has the option of automated false coloring (see Methods section for details), which is shown in Figures 4, 5, 6.

### Extensions of Z-LSR method

In the Z-LSR method is efficient, and enhances signal to noise by specifically highlighting spectral differences in the sample. In this method, a single parameter, the slope of a linear fit, is used to characterize the spectral dimension for each x-y point in the cell. The characterization of a very complicated spectral dimension by the slope of a fitted line is a poor approximation, and could, in principal, be extended by use of a higher-order functional form. For example, the use of peak position and height, or expansion in terms of more appropriate basis functions, such as wavelets, represents an interesting avenue of exploration. Wavelets have been used specifically for noise filtering in single Raman spectra [11] but not for peak finding in order to generate automated spectral contrast. Another possible extension is to use machine learning techniques to map spectral information to specific chemical information. Such an approach would require significant training data, however, which is not currently available. The current method thus represents a proof of concept that even simple functional forms can be used to characterize points in a cell by their spectral content. Note also that the absolute intensity is lost by normalization. What is gained is a boost in signal to noise because the background intensity is effectively set to zero by the z-score. We have not explored using the variation in the absolute intensity as a weight in reconstruction of the final image.

## Conclusions

In this study, we have presented two approaches to image processing of raw Raman microscopy images of un-stimulated mouse macrophage cells. The first approach is to use statistical methods to rapidly highlight spectral distributions in the cells using Z-LSR. The complementary approach is to use eigenspace processing or similar methods of multivariate analysis. These are typically slow, but can be used for extracting the component spectra. For such multivariate methods, the assignment of component spectra as noise or signal remains a problem that is difficult to automate. Our approach was to rank the eigenvectors by their RMSD in an attempt to automated at least the eigenvector ranking process in the reconstruction of Raman data following SVD, albeit with the high computational cost that is inherent to SVD-based approaches. As a complementary method, the Z-LSR technique hybridizes other simpler approaches while dramatically improving run-time requirements and providing an intrinsically clear picture of signals within the Raman microscopy data. It can be implemented faster than the time required to acquire the actual Raman data for close to real-time processing during Raman imaging, and it can be used in combination with SVD by pre-screening large amounts of data to determine specific data sets of interest, which may then be processed by SVD or other intensive (and slow) methods. The cells used in these experiments were unstimulated mouse macrophage cells, but the Z-LSR approach is fully automatable and inherently normalizes and removes bias from the datasets, which is a necessary step towards the creation of a Raman spectra database for comparing different cellular conditions such as stimulation of the immune response in the macrophages.

## Methods

### Overview

**Figure 7:**
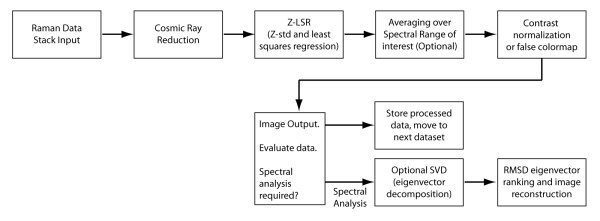
**Overview of Raman dataset processing**.

Two different types of processing were explored in the present study: a hybrid method utilizing Z-Score standardization and least squares regression (Z-LSR), as well as the established method of Singular Value Decomposition (SVD) and reconstruction by eigenvectors. Images must also be pre-processed to remove spurious signals due to cosmic rays. Image post-processing includes contrast normalization and optionally false color-mapping, or bicubic interpolation if required [12], as illustrated in figure 7.

### Raman Data acquisition

Cultured HeLa cells were grown in Dulbecco's modified Eagle's medium (DMEM; Nissui) supplemented with 10% fetal calf serum, 2 mM glutamine, 100 units/mL penicillin, and 100 μg/mL streptomycin, in a humidified atmosphere (5% CO2, 95% air) at 37°C. 1 mm thick, 25 mm diameter quartz substrates were used as cell culture substrates for low background signal during Raman microscopy. Raman imaging was performed using the 532 nm excitation laser of a confocal Raman micro-spectroscopy system (Raman-11, Nanophoton). The excitation source was a semiconductor laser operating at 532 nm, with a power of up to 300 mW. The laser beam was focused into a line illumination pattern and irradiated the sample via a water-immersion objective lens (Nikon, 100X, 1.0NA). Scattered light was collected with the same objective and guided into a Czerny-Turner-type spectrometer with a focal length of 50 cm, and the final spectroscopic data was collected by an electrically cooled CCD camera (Princeton Instruments, Pixis 400). The overall spectral resolution was 1.6 cm^-1^, and optical resolution was approximately 350 nm. Due to the line illumination configuration of the beam, laser power or intensity at the focus was difficult to measure but was between 0.1 and 2.5 mW per square micrometer for all sample images.

Spectral data wavenumber calibration was evaluated by collecting the Raman spectra of ethanol and comparing peak positions to ethanol spectra in the Spectral Database for Organic Compounds (SDBS) [13] with peaks matching within three wavenumbers as follows: (comparison shows measured/database peak pairs in cm^-1^) 1094/1097, 1452/1455, 2877/2876, 2927/2927, and 2971/2973.

### Cosmic Ray Reduction

There are numerous sources of noise in acquired Raman datasets. These include the granular nature of the low photon numbers, thermal noise in the detection, background Raman or fluorescence signals from quartz substrates along with other sources of noise that are more or less evenly distributed throughout the dataset. The most corrupting noise type in the datasets tends to be due to cosmic rays impinging on the detector. The cosmic rays usually corrupt only a single pixel or small group of pixels, replacing the data value with an erroneous value often an order of magnitude higher than the Raman data values. The cosmic ray corruption therefore appears as a spurious delta function isolated in both x-y dimension and spectral value. The cosmic ray reduction pre-processing step removes these outlier values that corrupt or wash out valid signal peak information corresponding to useful biological macrophage detail. Cosmic rays are an inevitable result of long exposure times, which are required in Raman microscopy, and there are many potential approaches to removing such corruption of the data. Keeping in mind the targets of automated data processing and scalability, we used a simple heuristic algorithm determined by the specific physical nature of the cosmic ray based noise, which primarily manifests as large deviations in pixel value but only affects isolated pixels. The algorithm is as follows: for a pixel in x-y, any wavenumber value which lies outside 3 standard deviations from the average for all wavenumbers in that pixel are deemed to be outliers and are assumed to be cosmic ray based corruption of the data. To remove such cosmic ray outliers, we compute the mean and variance. After the mean and variance is determined, if the deviation for any wavenumber is above the chosen threshold (typically 3 standard deviations) then we consider it a cosmic ray. Cosmic rays are replaced by the last value, which lay within the threshold (i.e. the last wavenumber value which was not a cosmic ray). This has the effect of replacing cosmic ray noise with local "trusted" data values, since cosmic rays typically only modify one or two adjacent pixels in the data stack.

### Standardized Regression (Z-LSR)

In an attempt to manage the aforementioned drawbacks of SVD, we applied statistical methods utilizing Z-Score standardization and least squares regression [12] which we refer to as the Z-LSR method. The computational complexity of this method is Ο(*MN*) such that *M *is the number of (*x*, *y*) points (e.g. 77 × 96) and *N *is the length of the w-dimension (e.g. 1,340) offering substantial performance improvement with similar (figure 2) or improved noise reduction compared with the SVD approach.

For each (*x*, *y*) position in the 3D Raman data stack, there is a w-axis spectral vector denoted as $\bar{s}$. The first step is to compute the z-score standardization $\bar{z}$ from the spectral vector $\bar{s}=\{s_{0},s_{1},s_{2}\cdot \cdot \cdot s_{N}\}$ as

(1) $$\bar{z}=\frac{\left(\bar{s}-\mu \right)}{\sigma }$$

(1)

where the mean (μ) and standard deviation (σ) are computed for the specific vector $\bar{s}$. The output vector $\bar{z}$ has the same length as the input vector $\bar{s}$. The second step is to compute the least squares regression slope *m *for each vector $\bar{z}=\{z_{0},z_{1},z_{2}\cdot \cdot \cdot z_{N}\}$. The slope associates a single scalar value with each standardized spectral vector, the hypothesis being that similar $\bar{z}$ vector spectra in the data stack will have similar slopes. The slope is then given by

(2) $$m=\left(\frac{N\sum_{i=1}^{N}iz_{i}-\sum_{i=1}^{N}i\sum_{i=1}^{N}z_{i}}{N\sum_{i=1}^{N}i^{2}-{\left(\sum_{i=1}^{N}i\right)}^{2}}\right)$$

(2)

The third and last step is to produce a new Z-LSR spectral vector $\bar{v}$ by multiplying all values in the vector $\bar{z}$ by their associated slope *m*. This process is repeated for all z-score vectors $\bar{z}$ until the Raman data stack has been replaced with all of the $\bar{v}$ vectors. Ultimately, the image representation is revealed by consistent regression trends multiplied by the standardized value.

### Automated Color-Mapping

The automated color-mapping was done by searching through the data to locate the strongest three peaks, for all spectral data. Red, green and blue channels were then assigned respectively on a per-pixel basis so that the pixel color corresponded to the relative strengths of the previously defined three peaks. This produced fully automated color imaging based on the strongest spectral information in the data. Due to the differences in sample composition and varying amounts of cell to substrate signal in different experiments, automated color-mapping produced different pseudocolor images for different data sets. This differences were determined predominantly by the ratio of the cell to substrate area in the dataset, since the strongest contribution to the spectra came from the background if there was little cellular material in the data set and vice versa for datasets where the substrate was covered by cells. We also experimented with mapping groups of peaks to each individual color channels, but have shown only color images where the top three peaks were mapped to a single color channel.

### SVD

SVD [14] begins with the construction of matrix *A_(N × M) _*such that n is the number of rows and m is the number of columns. This matrix is then decomposed into three matrices defined by the equation: $A_{N\times M}=U_{N\times N}\cdot S_{N\times M}\cdot V_{M\times M}^{T}$ such that *U *represents the left singular vectors, *S *represents the singular values in non-increasing order along the diagonal, and the transpose of *V *represents the right singular vectors. In the present work, the matrix *A *was constructed from the raw Raman spectra. For example, the data set depicted in figure 1 contains the dimensions 77 × 96 × 1340; therefore, Matrix *A *will become a 2D representation of this data set with 7,392 columns (i.e. the X/Y planes are constructed as a 1D piecewise linear array) and 1,340 rows representing the spectral data: *A_(1340 × 7392)_*. The computation of the SVD means solving for the eigenvalues and eigenvectors of the matrices *AA^T ^*(i.e. the columns of *U*) and *A^T^A *(i.e. the columns of *V*). The concept of orthogonality can be expressed by the equation: *e^T^e *= *I *such that *e *is an eigenvector and *I *is the identity matrix. This is the underlying reason why we can separate the Raman spectra into 'noise' and 'signal'.

Identifying the eigenspectra of interest was done by selecting the eigenspectra with the highest root-mean-squared deviation (RMSD). Given a vector $\bar{x}$ with *N *spectral values, such that one vector exists for each eigenspectra reconstruction, we sorted the eigenvector index position using its corresponding RMSD value defined as $S_{RMSD}=\sqrt{x_{1}^{2}+x_{2}^{2}+x_{3}^{2}+\cdot \cdot \cdot +x_{n}^{2}/N}$, and sorted eigenvectors in descending RMSD value. This approach empirically performed well because noisy eigenspectra oscillate very near to zero, whereas signals arising from bio-molecules of interest, have positive intensities (e.g., figure 3C).

## Competing interests

The authors declare that they have no competing interests.

## Authors' contributions

YK prepared macrophage cells. NIS acquired Raman images. KM and NIS analyzed raw Raman data. RJM developed and implemented computational methods. DMS and NIS conceived the study. RJM, DMS, and NIS wrote the manuscript. All authors participated in discussions, and reviewed and approved the final manuscript version.

## Supplementary Material

Additional file 1 **Overview of Raman scattering**. A brief overview of the Raman scattering process is useful to help understand the methods of acquiring data in this manuscript. The slit-scanning geometry is not shown, but is discussed in Hamada et al 2008 [1]. The Raman scattering effect is shown schematically in figure S1. In Raman scattering detection used for these experiments, light of 532 nm wavelength hits the target cell, producing scattering of the same wavelength (elastic scattering), as well as scattering where the light wavelength is shifted, either to a longer or shorter wavelength. This type of scattering is inelastic, since energy is either absorbed by or removed from the target molecules, thereby changing the vibrational state of the molecule. For molecules in the ground state, the emitted photon can be of longer wavelength (i.e. red-shifted) by an amount corresponding to the degree of energy deposited in the molecular vibrations of the sample. This is how the Raman scattering effect occurs, and since the shift corresponds to molecular vibrational states, the overall molecular composition of the sample can in principle be determined. The Raman scattering effect relies on the Stokes shift (related to the difference between incident photon wavelength and emitted photon wavelength). If the molecules are already in a vibrational state, they can be moved to a ground state and emit a photon of shorter wavelength (i.e. blue-shifted), which is known as Anti-Stokes Raman scattering. Both Stokes and Anti-Stokes Raman measurements can provide similar but somewhat complementary information. Unless specified, Raman scattering refers to Stokes not Anti-Stokes scattered photons. Click here for file
