# Supplementary figures and images for: Automated processing of label-free Raman microscope images of macrophage cells with standardized regression for high-throughput analysis
Source: Immunome Res. 2010 Nov 19;6:11. doi: 10.1186/1745-7580-6-11 (PMC2995782; doi:10.1186/1745-7580-6-11)

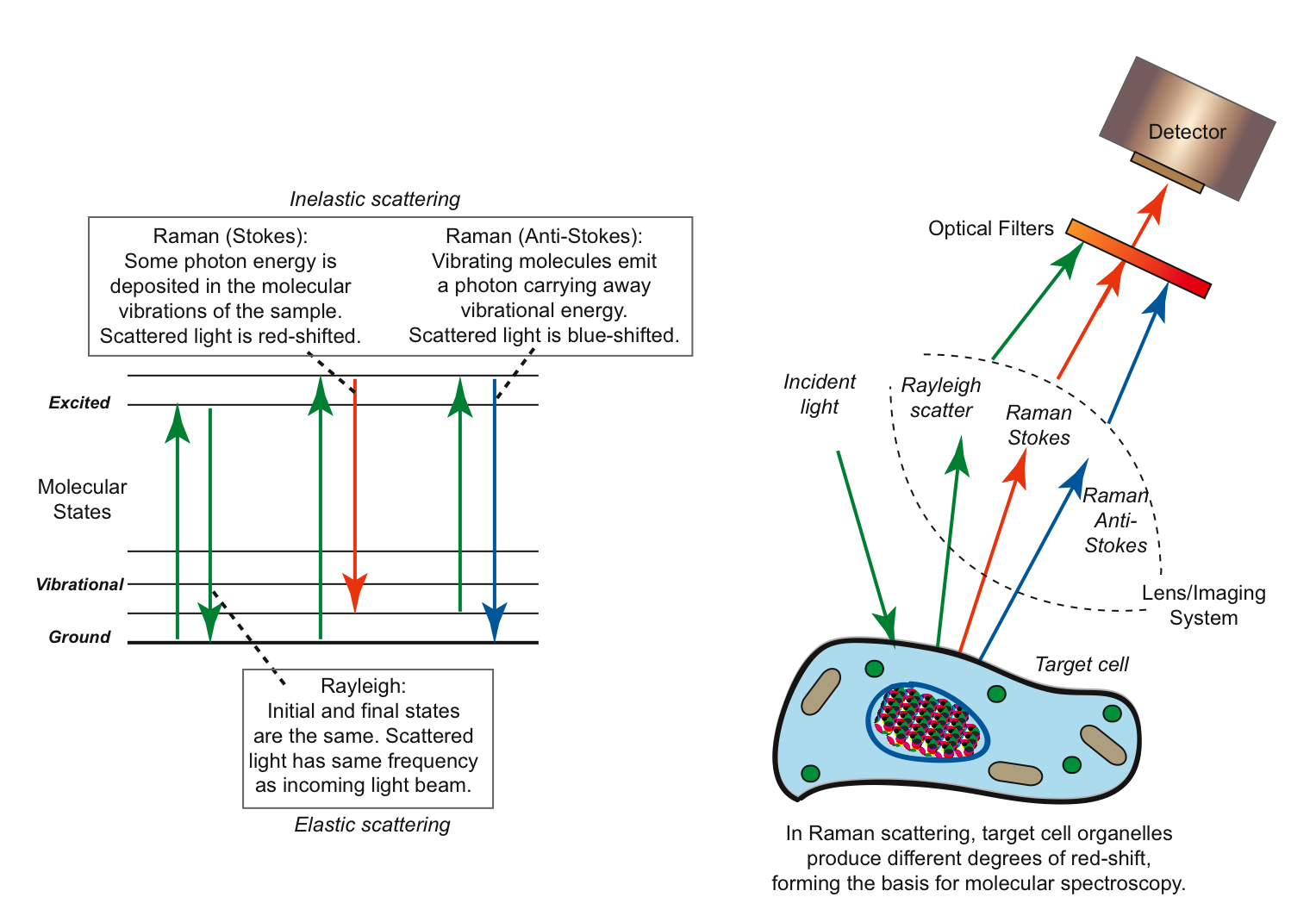

Supplement: Additional file 1 — Overview of Raman scattering. A brief overview of the Raman scattering process is useful to help understand the methods of acquiring data in this manuscript. The slit-scanning geometry is not shown, but is discussed in Hamada et al 2008 [1]. The Raman scattering effect is shown schematically in figure S1. In Raman scattering detection used for these experiments, light of 532 nm wavelength hits the target cell, producing scattering of the same wavelength (elastic scattering), as well as scattering where the light wavelength is shifted, either to a longer or shorter wavelength. This type of scattering is inelastic, since energy is either absorbed by or removed from the target molecules, thereby changing the vibrational state of the molecule. For molecules in the ground state, the emitted photon can be of longer wavelength (i.e. red-shifted) by an amount corresponding to the degree of energy deposited in the molecular vibrations of the sample. This is how the Raman scattering effect occurs, and since the shift corresponds to molecular vibrational states, the overall molecular composition of the sample can in principle be determined. The Raman scattering effect relies on the Stokes shift (related to the difference between incident photon wavelength and emitted photon wavelength). If the molecules are already in a vibrational state, they can be moved to a ground state and emit a photon of shorter wavelength (i.e. blue-shifted), which is known as Anti-Stokes Raman scattering. Both Stokes and Anti-Stokes Raman measurements can provide similar but somewhat complementary information. Unless specified, Raman scattering refers to Stokes not Anti-Stokes scattered photons. [file 1745-7580-6-11-S1.PNG]
